# Synthesis and Bioactivity of a Cyclopolypeptide from Caribbean Marine Sponge

**DOI:** 10.22037/ijpr.2020.15405.13075

**Published:** 2020

**Authors:** Rajiv Dahiya, Stacy Rampersad, Terry G. Ramnanansingh, Komalpreet Kaur, Ramninder Kaur, Rita Mourya, Suresh V. Chennupati, Richard Fairman, Nigel K. Jalsa, Ajay Sharma, Shivkanya Fuloria, Neeraj Kumar Fuloria

**Affiliations:** a *Laboratory of Peptide Research and Development, School of Pharmacy, Faculty of Medical Sciences, The University of the West Indies, St. Augustine, Trinidad and Tobago. *; b *Department of Pharmaceutical Chemistry, GHG Khalsa College of Pharmacy, Gurusar Sadhar, Ludhiana, Punjab, India. *; c *School of Pharmacy, College of Medicine and Health Sciences, University of Gondar, Gondar, Ethiopia. *; d *Department of Pharmacy, College of Medical and Health Sciences, Wollega University, Nekemte, Ethiopia. *; e *Department of Chemistry, Faculty of Science and Technology, The University of the West Indies, St. Augustine, Trinidad and Tobago.*; f *Department of Pharmacognosy and Phytochemistry, School of Pharmaceutical Sciences, Delhi Pharmaceutical Sciences and Research University, New Delhi, India. *; g *Department of Pharmaceutical Chemistry, Faculty of Pharmacy, AIMST University, Semeling, Bedong, Kedah, Malaysia.*

**Keywords:** Rolloamide A, Cyclic heptapeptide, Solution-phase peptide synthesis, Cyclization, Antibacterial activity, Antifungal activity, Anthelmintic activity

## Abstract

Synthesis of a natural proline-rich cyclopolypeptide - rolloamide A was carried out by coupling of tri- and tetrapeptide units Boc-Phe-Pro-Val-OMe and Boc-Pro-Leu-Pro-Ile-OMe after proper deprotection at carboxyl and amino terminals using carbodiimide chemistry in alkaline environment followed by cyclization of linear heptapeptide segment in the presence of base. The structure of synthesized peptide was confirmed by spectral techniques including FTIR, ^1^H NMR, ^13^C NMR, MS analyses. Newly synthesized peptide was subjected to biological screening against pathogenic microbes and earthworms. Cyclopeptide **8** possessed promising activity against pathogenic fungi *Candida albicans* (ZOI: 24 mm, MIC: 6 μg/mL) and Gram-negative bacteria *Pseudomonas aeruginosa *(ZOI: 27 mm, MIC: 6 μg/mL) and *Klebsiella pneumoniae* (ZOI: 23 mm, MIC: 12.5 μg/mL), in comparison to reference drugs – griseofulvin (ZOI: 20 mm, MIC: 6 μg/mL) and ciprofloxacin (ZOI: 25 mm, MIC: 6 μg/mL/ZOI: 20 mm, MIC: 12.5 μg/mL). Also, newly synthesized heptacyclopeptide exhibited potent anthelmintic activity against earthworms *Megascoplex konkanensis,* *Pontoscotex corethruses*, and *Eudrilus* species (MPT/MDT ratio – 8.22-16.02/10.06-17.59 min), in comparison to standard drugs - mebendazole (MPT/MDT ratio – 10.52-18.02/12.57-19.49 min) and piperazine citrate (MPT/MDT ratio – 12.38-19.17/13.44-22.17 min).

## Introduction

Since decades, marine environment is acquiring more interest as a source of new bioactive compounds. Marine organism’s derived bioactive peptides are considered a promising group of natural substances with pharmacological properties. In spite of many breakthroughs in the synthesis and delivery of peptides and proteins, the complete exploration of these bio-macromolecules is still a challenge to researchers and pharmaceutical scientists (1). Among marine animals, sponges have attracted the great interest of pharmacologists, chemists, and biochemists as a rich source of peculiar antimicrobial compounds especially cyclopolypeptides (2, 3). Till now, a significant number of cyclic peptides has been isolated from the marine resources including sponges with antimicrobial (4, 5), cytotoxic (6-9), antifouling (10), anti-tubercular (11), anti-dormant, and HIV-inhibitory potential (12, 13). Due to their therapeutic abilities, peptides have received growing interest and involved in treating novel targets for certain disease conditions, including Alzheimer’s disease, diabetes mellitus type 2, and obesity (14-16). However, peptides, being biomacromolecules, also exhibit various challenges such as limited water solubility, stability aspects, as well as structural and synthesis complexities, limiting their full exploitation in drug development (17).

Peptides in a circular form provide a promising scaffold for the development of a novel drug class owing to their adjustable and expandable ability to bind a wide range of target molecules. The moderate size and diverse functional groups of peptides ensure that the contact area is large enough to provide high selectivity, and their potential to form multiple hydrogen bonds can lead to strong binding affinity. In addition, cyclization of peptides generates structural and functional features that are critical for their use as pharmaceutical agents. The structural constraints provided by cyclization help to resist degradation by proteases in the blood, thereby increasing their serum stability. Cyclization of peptides also facilitates passage through the cell membrane, thus broadening the potential use of cyclic peptides beyond extracellular targets to include intracellular targets. Because of such favorable features, various cyclic peptides from natural sources and their derivatives have been exploited for biomedical and other purposes (18-21). Further, sponge-derived cyclic peptides play a vital role in drug design and have provided significant promise for future endeavors. Peptide drugs can be less harmful, after acting on target molecules, as they will disappear rapidly by proteolytic degradation. The degradation products are simply amino acids and would not have toxicity. Peptides can work on their targets very selectively, as the interaction with the targets is very specific compared to small molecules. Usually, cyclic peptides show better biological activity compared to their linear counterparts due to the conformational rigidity. The rigidity of cyclic peptides decreases the entropy term of the Gibbs free energy, therefore allowing the enhanced binding toward target molecules, or receptor selectivity. Considering these strengths, it is not surprising that there are many peptide drugs available in the market (22). 

Nowadays, proline-rich peptides have received special attention that includes a large and heterogeneous group of small-medium sized structures characterized by the presence of proline residues, often constituting peculiar sequences. This feature confers them a typical structure that determines the various biological functions endowed by these molecules. In particular, the left-handed-polyproline-II helix is essential for the expression of the antimicrobial, immunomodulatory, antioxidant properties and to finely modulate protein-protein interactions, thus playing crucial roles in many cell signal transduction pathways (23). Further, the proline-rich peptides represent a potentially new class of cell-permeant peptides for intracellular delivery of protein cargo and antimicrobial peptides may represent a rich source of templates for designing cell-permeant peptides (24). Because of their size and complexity, proline-rich cyclic peptides occupy a crucial chemical space in drug discovery that may provide useful scaffolds for modulating more challenging biological targets, such as protein-protein interactions and allosteric binding sites. These are formed by linking one end of the peptide and the other with an amide bond or other chemically-stable bonds. Some of them are used in the clinic, *e.g.* gramicidin S and tyrocidine with bactericidal activity, while others are in clinical trials, *e.g.* dehydrodidemnin B, and most of them are associated with wide array of pharmacological properties which prompted various research groups to synthesize these bioactives in the laboratory using solution-phase and solid-phase techniques (25-37).

Rolloamides are sponge-originated cytotoxic cyclopolypeptides from Caribbean region. The cyclic heptapeptide rolloamide A differs from rolloamide B in having three alternate L-proline units with *cis* geometry, whereas the latter contains two L-proline units, one with *cis *and another one with *trans* geometry, in addition to L-serine moiety. The synthesis of cyclopolypeptide rolloamide B has been reported by diverse research groups in literature employing solution-phase benzotriazole-mediated peptide synthesis technique and isobutyl chloroformate (IBCF) mediated direct coupling reaction (38,39). Rolloamide B was found to possess strong activity against several Gram-negative bacteria along with high antifungal potency.

Rolloamide A was found to exhibit significant growth suppression against a panel of human tumor cell lines including prostate, breast, ovarian, glioma, renal cancer cells with IC_50_ in range of 0.17-5.8 µM. Maximum bioactivity was displayed against Ovarian OVICAR3 cell line, Breast MDA468, and MDA435 cell lines with IC_50_ values of 0.17, 0.38, and 0.40 µM. The natural cycloheptapeptide, rolloamide A, was isolated from the Dominican marine sponge *Eurypon laughlini*, the complex structure of which was confirmed by a combination of spectroscopic analyses and chemical degradation (40).

In continuation of our investigation on novel biologically active cyclic peptides of natural origin (41-47), we focused our attention on the synthesis of a natural proline-rich cycloheptapeptide of marine origin. In the present study, we describe the solution-phase synthesis, structure elucidation, and bioevaluation of rolloamide A for antibacterial, antifungal, and anthelmintic properties.

## Experimental

*Materials and methods*

Melting point was determined by open capillary method. L-Amino acids, coupling agents, and other chemicals used were obtained from Spectrochem Limited (Mumbai, India). IR spectra, ^1^H / ^13^C NMR spectra were recorded on Shimadzu 8700 FTIR spectrophotometer (Shimadzu, Japan) and Bruker AC NMR spectrometer (300 MHz), (Bruker, USA). Mass spectrum was recorded on a JMS-DX 303 mass spectrometer (Jeol, Japan) operating at 70 eV using the fast atom bombardment technique (FAB MS). Elemental analysis was performed on Vario EL III elemental analyzer (Elementar) and optical rotation was measured on automatic polarimeter (Optics Tech) and purity of all compounds was checked by TLC on precoated silica gel G plates.

*General method for the synthesis of dipeptide fragments [* ***1-3*** *]*

At 0 °C, *N*-methylmorpholine (NMM, 2.3 mL, 0.021 mol) was added to amino acid methyl ester hydrochloride (0.01 mol) previously dissolved in dichloromethane (DCM, 25 mL) and resulting reaction mixture was stirred for 25 min. The 1-ethyl-3-(3-dimethylaminopropyl) carbodiimide hydrochloride (EDC.HCl) (1.92 g, 0.01 mol) and 1-hydroxybenztriazole (HOBt, 1.34 g, 0.01 mol) were mixed with Boc-amino acid (0.01 mol) in dichloromethane (25 mL) with stirring and finally, added to the above reaction mixture. After 24 h, the final reaction mixture was filtered and the filtrate was washed with 5% NaHCO_3 _and saturated NaCl solutions. The organic layer was dried over anhydrous Na_2_SO_4_, filtered and evaporated in vacuum. The crude product was recrystallized from a mixture of chloroform and petroleum ether. 

*tert-Butyloxycarbonyl-phenylalanyl-proline methyl ester [* ***1*** *]*

Semisolid mass, Yield 87.6%, [*α*]_D_ –44.3°, R_f_ - 0.59; IR (CHCl_3_):* v *3122 (m, -NH str, amide), 3066-3062 (w, -CH str, aromatic ring), 2998, 2993 (m, -CH str, cyclic CH_2_ and CH), 2927 (m, -CH str, asym, CH_2_), 2848 (m, -CH str, sym, CH_2_), 1751 (s, -C=O str, ester), 1674, 1633 (s, -C=O str, 3° and 2° amide), 1559, 1429 (m, skeletal bands, aromatic ring), 1532 (m, -NH bend, 2° amide), 1390, 1368 (m, -CH bend, *tert*-butyl group), 1268 (s, C−O str, ester), 715, 689 (s, -CH bend, oop, aromatic ring) cm^-1^; ^1^H NMR (300 MHz, CDCl_3_): *δ* 7.48 (2H, t, *J* = 7.25 Hz, *m*-H’s, Phe), 6.92 (1H, t, *J* = 7.3 Hz, *p*-H, Phe), 6.85 (2H, d, *J* = 7.2 Hz, *o*-H’s, Phe), 6.45 (1H, br. s, -NH), 4.78 (1H, q, *J* = 5.6 Hz, *α*-H, Phe), 4.26 (1H, t, *J* = 8.2 Hz, *α*-H, Pro), 3.77 (2H, t, *J* = 7.9 Hz, *δ*-H’s, Pro), 3.65 (3H, s, OCH_3_), 3.03 (2H, d, *J* = 5.9 Hz, *β*-H’s, Phe), 2.07-2.03 (2H, q, *β*-H’s, Pro), 1.98-1.93 (2H, m, *γ*-H’s, Pro), 1.54 (9H, s, *tert*-butyl group) ppm; ^13^C NMR (CDCl_3_, 300 MHz): *δ* 169.9, 168.8 (2C, C=O, Phe and Pro), 152.3 (C=O, Boc), 133.4 (*γ*-C, Phe), 130.6 (2C, *o*-C’s, Phe), 129.2 (2C, *m*-C’s, Phe), 128.0 (*p*-C, Phe), 79.5 (*α*-C, Boc), 58.9 (*α*-C, Pro), 53.5 (OCH_3_), 52.4 (*α*-C, Phe), 46.5 (*δ*-C, Pro), 38.9, 31.7 (2C, *β*-C’s, Phe and Pro), 28.2 (3C, *β*-C’s, Boc), 23.4 (*γ*-C, Pro) ppm; Anal. Calcd. for C_20_H_28_N_2_O_5_: C, 63.81; H, 7.50; N, 7.44. Found: C, 63.79; H, 7.49; N, 7.47%.

*tert-butyloxycarbonyl-prolyl-leucine methyl ester [* ***2*** *]*

Semisolid mass, Yield 75.2%, [*α*]_D_: –111.2°, R_f_ - 0.68; IR (CHCl_3_):* v *3125 (m, -NH str, amide), 2996, 2992 (m, -CH str, cyclic CH_2_ and CH), 2969-2965, 2925 (m, -CH str, asym, CH_3 _and CH_2_), 2849 (m, -CH str, sym, CH_2_), 1754 (s, -C=O str, ester), 1672, 1635 (s, -C=O str, 3° and 2° amide), 1529 (m, -NH bend, 2° amide), 1388, 1369 (m, -CH bend, *tert*-butyl group), 1383, 1362 (m, -CH bend, *iso*-propyl group), 1272 (s, C−O str, ester) cm^-1^; ^1^H NMR (300 MHz, CDCl_3_): *δ* 6.65 (1H, br. s, -NH), 4.37 (1H, q, *J* = 6.7 Hz, *α*-H, Leu), 4.05 (1H, t, *J* = 7.8 Hz, *α*-H, Pro), 3.63 (3H, s, OCH_3_), 3.23 (2H, t, *J* = 7.85 Hz, *δ*-H’s, Pro), 2.55 (2H, q, *β*-H’s, Pro), 1.95-1.89 (2H, m, *γ*-H’s, Pro), 1.49 (9H, s, *tert*-butyl group), 1.46 (2H, t, *J* = 6.9 Hz, *β*-H’s, Leu), 1.43-1.38 (1H, m, *γ*-H, Leu), 0.95 (6H, d, *J* = 6.45 Hz, *δ*-H’s, Leu) ppm; ^13^C NMR (CDCl_3_, 300 MHz): *δ* 175.8, 169.9 (2C, C=O, Leu and Pro), 157.3 (C=O, Boc), 80.6 (*α*-C, Boc), 62.3 (*α-*C, Pro), 52.1 (OCH_3_), 49.4 (*α-*C, Leu), 46.7 (*δ*-C, Pro), 42.9 (*β-*C, Leu), 28.4 (*β*-C, Pro), 27.6 (3C, *β*-C’s, Boc), 26.2 (*γ*-C, Leu), 24.7 (2C, *δ*-C’s, Leu), 23.4 (γ-C, Pro) ppm; Anal. Calcd. for C_17_H_30_N_2_O_5_: C, 59.63; H, 8.83; N, 8.18. Found: C, 59.65; H, 8.85; N, 8.15%.

^t^ *Butyloxycarbonyl-prolyl-isoleucine methyl ester [* ***3*** *]*

Semisolid mass, Yield 77.9%, [*α*]_D_: –79.3°, R_f_ - 0.52; IR (CHCl_3_):* v *3122 (m, -NH str, amide), 2997-2993 (m, -CH str, cyclic CH_2_ and CH), 2967, 2927 (m, -CH str, asym, CH_3 _and CH_2_), 2870, 2845 (m, -CH str, sym, CH_3_ and CH_2_), 1751 (s, -C=O str, ester), 1669, 1633 (s, -C=O str, 3° and 2° amide), 1528 (m, -NH bend, 2° amide), 1386, 1368 (m, -CH bend, *tert*-butyl group), 1270 (s, C−O str, ester) cm^-1^; ^1^H NMR (300 MHz, CDCl_3_): *δ* 5.97 (1H, br. s, -NH), 4.10 (1H, t, *J* = 7.6 Hz, *α*-H, Ile), 4.06 (1H, t, *J* = 7.85 Hz, *α*-H, Pro), 3.51 (3H, s, OCH_3_), 3.20 (2H, t, *J* = 7.9 Hz, *δ*-H’s, Pro), 2.57 (2H, q, *β*-H’s, Pro), 2.05-1.99 (1H, m, *β*-H, Ile), 1.92-1.87 (2H, m, *γ*-H’s, Pro), 1.70-1.65 (2H, m, *γ*-H’s, Ile), 1.47 (9H, s, *tert*-Butyl group), 0.95 (3H, t, *J* = 7.25 Hz, *δ*-H’s, Ile), 0.89 (3H, d, *J* = 6.55 Hz, *γ’*-H’s, Ile) ppm; ^13^C NMR (CDCl_3_, 300 MHz): *δ* 172.4, 169.5 (2C, C=O, Ile and Pro), 156.5 (C=O, Boc), 79.9 (*α*-C, Boc), 60.7 (*α*-C, Pro), 56.6 (*α*-C, Leu), 54.5 (OCH_3_), 46.9 (*δ*-C, Pro), 37.7 (*β*-C, Ile), 28.9 (*β*-C, Pro), 27.7 (3C, *β*-C’s, Boc), 24.1 (*γ*-C, Ile), 23.6 (*γ*-C, Pro), 15.2 (*γ’*-C, Ile), 10.3 (*δ*-C, Ile) ppm; Anal. Calcd. for C_17_H_30_N_2_O_5_: C, 59.63; H, 8.83; N, 8.18. Found: C, 59.62; H, 8.80; N, 8.16%.

*Method for the deprotection of dipeptides [* ***1, 2*** *] at carboxyl end*

To a solution of the dipeptide (**1**, 3.76 g, 0.01 mol) in THF : H_2_O (1:1, 36 mL), LiOH (0.36 g, 0.015 mol) was added at 0 °C. The mixture was stirred at RT for 1 h and then acidified to pH 3.5 with 1 N H_2_SO_4_. The aqueous layer was extracted with Et_2_O (3 × 25 mL). The combined organic extracts were dried over anhydrous Na_2_SO_4_ and concentrated under reduced pressure. The crude product was crystallized from methanol and ether to get deprotected dipeptide Boc-Phe-Pro-OH [**1a**]. Similarly, dipeptide **2** (3.42 g, 0.01 mol) was hydrolyzed under alkaline conditions to obtain Boc-Pro-Leu-OH [**2a**]. 

*tert-Butyloxycarbonyl-phenylalanyl-proline-OH [* ***1a*** *]*

Semisolid mass, Yield 66.8%, [*α*]_D_ –73.7°, R_f_ - 0.46; IR (CHCl_3_):* v *3125 (m, -NH str, amide), 3290-2493 (m, -OH str, COOH), 3065-3061 (w, -CH str, aromatic ring), 2998-2995 (m, -CH str, cyclic CH_2_ and CH), 2929 (m, -CH str, asym, CH_2_), 2847 (m, -CH str, sym, CH_2_), 1705 (s, C=O str, COOH), 1677, 1631 (s, -C=O str, 3° and 2° amide), 1556, 1427 (m, skeletal bands, aromatic ring), 1411 (m, -OH def, COOH), 1535 (m, -NH bend, 2° amide), 1389, 1366 (m, -CH bend, *tert*-butyl group), 718, 685 (s, -CH bend, oop, aromatic ring) cm^-1^; ^1^H NMR (300 MHz, CDCl_3_): *δ* 10.46 (1H, br. s, OH, COOH), 7.52 (2H, t, *J* = 7.3 Hz, *m*-H’s, Phe), 6.95 (1H, t, *J* = 7.3 Hz, *p*-H, Phe), 6.82 (2H, d, *J* = 7.15 Hz, *o*-H’s, Phe), 6.46 (1H, br. s, -NH), 5.77 (1H, q, *J* = 5.65 Hz, *α*-H, Phe), 4.41 (1H, t, *J* = 8.15 Hz, *α*-H, Pro), 3.79 (2H, t, *J* = 7.85 Hz, *δ*-H’s, Pro), 3.09 (2H, d, *J* = 5.95 Hz, *β*-H’s, Phe), 2.09-2.04 (2H, q, *β*-H’s, Pro), 1.96-1.92 (2H, m, *γ*-H’s, Pro), 1.56 (9H, s, *tert*-butyl group) ppm; Anal. Calcd. for C_19_H_26_N_2_O_5_: C, 62.97; H, 7.23; N, 7.73. Found: C, 62.96; H, 7.22; N, 7.75%.

*tert-butyloxycarbonyl-prolyl-leucine-OH [* ***2a*** *]*

Semisolid mass, Yield 69.5%, [*α*]_D_: –57.9°, R_f_ - 0.78; IR (CHCl_3_):* v *3288-2498 (m, -OH str, COOH), 3122 (m, -NH str, amide), 2995, 2989 (m, -CH str, cyclic CH_2_ and CH), 2968-2963, 2928 (m, -CH str, asym, CH_3 _and CH_2_), 2852 (m, -CH str, sym, CH_2_), 1709 (s, C=O str, COOH), 1676, 1633 (s, -C=O str, 3° and 2° amide), 1526 (m, -NH bend, 2° amide), 1411 (m, -OH def, COOH), 1389, 1366 (m, -CH bend, *tert*-butyl group), 1382, 1360 (m, -CH bend, *iso*-propyl group) cm^-1^; ^1^H NMR (300 MHz, CDCl_3_): *δ* 12.49 (1H, br. s, OH, COOH), 6.72 (1H, br. s, -NH), 4.47 (1H, q, *J* = 6.65 Hz, *α*-H, Leu), 3.89 (1H, t, *J* = 7.85 Hz, *α*-H, Pro), 3.25 (2H, t, *J* = 7.9 Hz, *δ*-H’s, Pro), 2.57 (2H, q, *β*-H’s, Pro), 1.98-1.92 (2H, m, *γ*-H’s, Pro), 1.89-1.82 (1H, m, *γ*-H, Leu), 1.57 (2H, t, *J* = 6.85 Hz, *β*-H’s, Leu), 1.47 (9H, s, *tert*-butyl group), 0.98 (6H, d, *J* = 6.45 Hz, *δ*-H’s, Leu) ppm; Anal. Calcd. for C_16_H_28_N_2_O_5_: C, 58.52; H, 8.59; N, 8.53. Found: C, 58.54; H, 8.58; N, 8.50%.

*Method for the deprotection of dipeptide [* ***3*** *] at amino end*

The dipeptide **3** (3.42 g, 0.01 mol) was dissolved in CHCl_3_ (15 mL) and treated with trifluoroacetic acid (CF_3_COOH, 2.28 g, 0.02 mol). The resulting solution was stirred at RT for 1 h, washed with saturated NaHCO_3_ solution (25 mL). The organic layer was dried over anhydrous Na_2_SO_4_ and concentrated under reduced pressure. The crude product was purified by crystallization from CHCl_3_ and petroleum ether (b.p. 40-60 °C) to get pure deprotected dipeptide Pro-Ile-OMe [**3a**].

*Prolyl-isoleucine methyl ester [* ***3a*** *]*

Semisolid mass, Yield 70.5%, [*α*]_D_: –103.7°, R_f_ - 0.77; IR (CHCl_3_):* v *3235, 3125 (m, -NH str), 2999-2994 (m, -CH str, cyclic CH_2_ and CH), 2965, 2929 (m, -CH str, asym, CH_3 _and CH_2_), 2872, 2843 (m, -CH str, sym, CH_3_ and CH_2_), 1748 (s, -C=O str, ester), 1630 (s, -C=O str, 2° amide), 1532 (m, -NH bend, 2° amide), 1268 (s, C−O str, ester) cm^-1^; ^1^H NMR (300 MHz, CDCl_3_): *δ* 6.96 (1H, br. s, -NH, Pro), 5.92 (1H, br. s, -NH), 4.16 (1H, t, *J* = 7.65 Hz, *α*-H, Ile), 3.54 (3H, s, OCH_3_), 3.49 (1H, t, *J* = 7.8 Hz, *α*-H, Pro), 2.77 (2H, t, *J* = 7.95 Hz, *δ*-H’s, Pro), 2.07-2.02 (1H, m, *β*-H, Ile), 1.88-1.83 (2H, q, *β*-H’s, Pro), 1.76-1.69 (2H, m, *γ*-H’s, Pro), 1.66-1.61 (2H, m, *γ*-H’s, Ile), 0.93 (3H, t, *J* = 7.2 Hz, *δ*-H’s, Ile), 0.86 (3H, d, *J* = 6.6 Hz, *γ’*-H’s, Ile) ppm; Anal. Calcd. for C_12_H_22_N_2_O_3_: C, 59.48; H, 9.15; N, 11.56. Found: C, 59.47; H, 9.16; N, 11.59%.

*General method for the synthesis of linear tri/tetra/heptapeptide fragments [* ***5-7*** *]*

Deprotected dipeptide unit Boc-Phe-Pro-OH (3.62 g, 0.01 mol) / Boc-Pro-Leu-OH (3.28 g, 0.01 mol) was dissolved in 25 mL of DCM/THF and solution was neutralized with 2.8 mL (0.021 mol) of triethylamine (TEA) at 0 °C and the resulting mixture was stirred for 25 min. Valine methyl ester hydrochloride (1.68 g, 0.01 mol)/dipeptide methyl ester Pro-Ile-OMe (2.42 g, 0.01 mol) was dissolved in 25 mL of DCM/THF and added to *N,N’*-diisopropylcarbodiimide (DIPC, 1.26 g, 0.01 mol) and HOBt (1.34 g, 0.01 mol). The resulting reaction mixture was added to the above mixture. Stirring was done for 24 h at room temperature. After 24 h, the final reaction mixture was filtered and the filtrate was washed with 5% NaHCO_3 _and saturated NaCl solutions. The organic layer was dried over anhydrous Na_2_SO_4_, filtered and evaporated in vacuum to obtain Boc-protected tri/tetrapeptide methyl esters Boc-Phe-Pro-Val-OMe **5 **/ Boc-Pro-Leu-Pro-Ile-OMe **6**. The crude products were purified from a mixture of chloroform and petroleum ether (b.p. 40-60 °C) followed by cooling at 0 °C. 

Linear heptapeptide fragment Boc-Phe-Pro-Val-Pro-Leu-Pro-Ile-OMe **7** was synthesized by following the same procedure *via* coupling of deprotected tripeptide unit Boc-Phe-Pro-Val-OH (4.62 g, 0.01 mol) and deprotected tetrapeptide unit Pro-Leu-Pro-Ile-OMe (4.53 g, 0.01 mol) using DIPC (1.26 g, 0.01 mol) as coupling agent and HOBt (1.34 g, 0.01 mol) as racemization suppressing agent. For synthesis of tetrapeptide and heptapeptide fragments, tetrahydrofuran (THF) was employed as solvent instead of DCM. Deprotection of the tripeptide unit Boc-Phe-Pro-Val-OMe **5** at carboxyl end was done by alkaline hydrolysis with lithium hydroxide by following the same procedure as opted for deprotection of dipeptide units **1 **and** 2**, while tetrapeptide unit Boc-Pro-Leu-Pro-Ile-OMe **6** was deprotected at amino terminal by using trifluoroacetic acid by following the same procedure as opted for deprotection of dipeptide unit **3**. 

^t^ *Butyloxycarbonyl-phenylalanyl-prolyl-valine methyl ester [* ***5*** *]*

Semisolid mass, Yield 82.7%, [*α*]_D_: +48.6°, R_f_ - 0.73, IR (CHCl_3_):* v *3126-3122 (m, -NH str, amide), 3064 (w, -CH str, aromatic ring), 2997-2992 (m, -CH str, cyclic CH_2_ and CH), 2925 (m, -CH str, asym, CH_2_), 1753 (s, -C=O str, ester), 1676, 1637-1632 (s, -C=O str, 3° and 2° amide), 1556, 1427 (m, skeletal bands, aromatic ring), 1535-1532 (m, -NH bend, 2° amide), 1389, 1366 (m, -CH bend, *tert*-butyl group), 1382, 1363 (m, -CH bend, *iso*-propyl group), 1272 (s, C−O str, ester), 711, 687 (s, -CH bend, oop, aromatic ring) cm^-1^; ^1^H NMR (300 MHz, CDCl_3_): *δ* 8.49 (1H, br. s, -NH, Val), 7.51 (2H, t, *J* = 7.3 Hz, *m*-H’s, Phe), 6.93 (1H, t, *J* = 7.25 Hz, *p*-H, Phe), 6.83 (2H, d, *J* = 7.15 Hz, *o*-H’s, Phe), 6.45 (1H, br. s, -NH, Phe), 4.78 (1H, t, *J* = 7.9 Hz, *α*-H, Val), 4.61 (1H, q, *J* = 5.65 Hz, *α*-H, Phe), 4.15 (1H, t, *J* = 8.15 Hz, *α*-H, Pro), 3.74 (2H, t, *J* = 7.85 Hz, *δ*-H’s, Pro), 3.64 (3H, s, OCH_3_), 3.12 (2H, d, *J* = 5.85 Hz, *β*-H’s, Phe), 2.68-2.64 (2H, q, *β*-H’s, Pro), 2.19-2.15 (1H, m, *β*-H’s, Val), 1.96-1.91 (2H, m, *γ*-H’s, Pro), 1.52 (9H, s, *tert*-butyl group), 0.85 (6H, d, *J* = 6.6 Hz, *γ*-H’s, Val) ppm; ^13^C NMR (CDCl_3_, 300 MHz): *δ* 172.2, 171.4, 170.1 (3C, C=O, Val, Pro and Phe), 152.7 (C=O, Boc), 133.0 (*γ*-C, Phe), 130.6 (2C, *m*-C’s, Phe), 128.2 (2C, *o*-C’s, Phe), 127.7 (*p*-C, Phe), 79.2 (*α-*C, Boc), 56.5 (*α*-C, Val), 56.0 (*α-*C, Pro), 55.2 (OCH_3_), 53.0 (*α*-C, Phe), 48.3 (*δ*-C, Pro), 38.2, 30.4 (2C, *β*-C’s, Phe and Val), 28.7 (3C, *β*-C’s, Boc), 26.4 (*β*-C, Pro), 24.1 (*γ*-C, Pro), 19.6 (2C, *γ*-C’s, Val) ppm; Anal. Calcd. for C_25_H_37_N_3_O_6_: C, 63.14; H, 7.84; N, 8.84. Found: C, 63.17; H, 7.82; N, 8.85%.

^t^ *Butyloxycarbonyl-prolyl-leucyl-prolyl-isoleucine methyl ester [* ***6*** *]*

Semisolid mass, Yield 83.9%, [*α*]_D_: –54.1°, R_f_ - 0.78, IR (CHCl_3_):* v *3128-3122 (m, -NH str, amide), 2999-2993 (m, -CH str, cyclic CH_2_ and CH), 2968-2963, 2925 (m, -CH str, asym, CH_3 _and CH_2_), 2869, 2847-2842 (m, -CH str, sym, CH_3_ and CH_2_), 1752 (s, -C=O str, ester), 1674-1669, 1638-1635 (s, -C=O str, 3° and 2° amide), 1527, 1523 (m, -NH bend, 2° amide), 1389, 1366 (m, -CH bend, *tert*-butyl group), 1381, 1360 (m, -CH bend, *iso*-propyl group), 1268 (s, C−O str, ester) cm^-1^; ^1^H NMR (300 MHz, CDCl_3_): *δ* 7.42 (1H, br. s, -NH, Ile), 7.34 (1H, br. s, -NH, Leu), 4.59 (1H, q, *J* = 6.65 Hz, *α*-H, Leu), 4.36 (1H, t, *J* = 7.7 Hz, *α*-H, Ile), 4.11 (1H, t, *J* = 7.9 Hz, *α*-H, Pro-1), 4.06 (1H, t, *J* = 7.8 Hz, *α*-H, Pro-2), 3.50 (3H, s, OCH_3_), 3.35 (2H, t, *J* = 7.8 Hz, *δ*-H’s, Pro-2), 3.19 (2H, t, *J* = 7.9 Hz, *δ*-H’s, Pro-1), 2.68 (2H, q, *β*-H’s, Pro-2), 2.53 (2H, q, *β*-H’s, Pro-1), 2.04-1.98 (1H, m, *β*-H, Ile), 1.94-1.86 (4H, m, *γ*-H’s, Pro-1 and Pro-2), 1.74 (2H, t, *J* = 6.85 Hz, *β*-H’s, Leu), 1.71-1.66 (2H, m, *γ*-H’s, Ile), 1.51 (9H, s, *tert*-butyl group), 1.46-1.39 (1H, m, *γ*-H, Leu), 0.99 (6H, d, *J* = 6.5 Hz, *δ*-H’s, Leu), 0.94 (3H, t, *J* = 7.3 Hz, *δ*-H’s, Ile), 0.87 (3H, d, *J* = 6.6 Hz, *γ’*-H’s, Ile) ppm; ^13^C NMR (CDCl_3_, 300 MHz): *δ* 172.9, 171.6 (2C, C=O, Pro-1 and Ile), 169.7, 168.2 (2C, C=O, Pro-2 and Leu), 156.6 (C=O, Boc), 79.7 (*α*-C, Boc), 60.6, 56.9, 55.3 (3C, *α*-C’s, Pro-1, Pro-2 and Ile), 53.7 (OCH_3_), 49.1 (*α*-C, Leu), 47.9, 46.5 (2C, *δ*-C’s, Pro-2 and Pro-1), 38.6, 36.1 (2C, *β*-C’s, Ile and Leu), 28.7 (*β*-C, Pro-1), 27.9 (3C, *β*-C’s, Boc), 26.6 (*β*-C, Pro-2), 24.2 (*γ*-C, Ile), 23.8 (*γ*-C, Pro-2), 22.9 (*γ*-C, Leu), 22.5 (*γ*-C, Pro-1), 21.9 (2C, *δ*-C’s, Leu), 15.1 (*γ’*-C, Ile), 9.5 (*δ*-C, Ile) ppm; Anal. Calcd. for C_28_H_48_N_4_O_7_: C, 60.85; H, 8.75; N, 10.14. Found: C, 60.88; H, 8.76; N, 10.17%.

^t^ *Butyloxycarbonyl-phenylalanyl-prolyl-valyl-prolyl-leucyl-prolyl-isoleucine methyl ester [* ***7*** *]*

Semisolid mass, Yield 70.7%, [*α*]_D_: –66.7°, R_f_ - 0.51, IR (CHCl_3_):* v *3129-3123 (m, -NH str, amide), 3067 (w, -CH str, aromatic ring), 2998-2994, 2993-2989 (m, -CH str, cyclic CH_2_ and CH), 2969-2965, 2927-2922 (m, -CH str, asym, CH_3 _and CH_2_), 2869-2866, 2848-2843 (m, -CH str, sym, CH_3_ and CH_2_), 1748 (s, -C=O str, ester), 1676-1672, 1637-1632 (s, -C=O str, 3° and 2° amide), 1559, 1425 (m, skeletal bands, aromatic ring), 1525, 1522 (m, -NH bend, 2° amide), 1387, 1368 (m, -CH bend, *tert*-butyl group), 1382, 1359 (m, -CH bend, *iso*-propyl group), 1269 (s, C−O str, ester), 714, 689 (s, -CH bend, oop, aromatic ring); ^1^H NMR (300 MHz, CDCl_3_): *δ* 9.23 (1H, br. s, -NH, Val), 8.84 (1H, br. s, -NH, Leu), 7.52 (2H, t, *J* = 7.25 Hz, *m*-H’s, Phe), 7.43 (1H, br. s, -NH, Ile), 6.92 (1H, t, *J* = 7.3 Hz, *p*-H, Phe), 6.85 (2H, d, *J* = 7.2 Hz, *o*-H’s, Phe), 6.45 (1H, br. s, -NH, Phe), 4.61 (1H, q, *J* = 5.7 Hz, *α*-H, Phe), 4.52 (1H, t, *J* = 7.85 Hz, *α*-H, Val), 4.44 (1H, q, *J* = 6.7 Hz, *α*-H, Leu), 4.38 (1H, t, *J* = 7.65 Hz, *α*-H, Ile), 4.17 (1H, t, *J* = 8.3 Hz, *α*-H, Pro-1), 4.07 (1H, t, *J* = 8.2 Hz, *α*-H, Pro-3), 3.84 (1H, t, *J* = 7.75 Hz, *α*-H, Pro-2), 3.72 (2H, t, *J* = 7.9 Hz, *δ*-H’s, Pro-1), 3.54 (3H, s, OCH_3_), 3.31 (2H, t, *J* = 7.9 Hz, *δ*-H’s, Pro-3), 3.15 (2H, d, *J* = 5.9 Hz, *β*-H’s, Phe), 3.08 (2H, t, *J* = 7.8 Hz, *δ*-H’s, Pro-2), 2.69-2.62 (6H, m, *β*-H’s, Pro-1–3), 2.09-2.05 (1H, m, *β*-H’s, Val), 2.03-1.98 (1H, m, *β*-H, Ile), 1.96-1.87 (6H, m, *γ*-H’s, Pro-1–3), 1.80 (2H, t, *J* = 6.9 Hz, *β*-H’s, Leu), 1.73-1.68 (2H, m, *γ*-H’s, Ile), 1.55 (9H, s, *tert*-butyl group), 1.52-1.45 (1H, m, *γ*-H, Leu), 1.07 (6H, d, *J* = 6.7 Hz, *γ*-H’s, Val), 0.99 (6H, d, *J* = 6.6 Hz, *δ*-H’s, Leu), 0.95 (3H, t, *J* = 7.25 Hz, *δ*-H’s, Ile), 0.88 (3H, d, *J* = 6.7 Hz, *γ’*-H’s, Ile) ppm; ^13^C NMR (CDCl_3_, 300 MHz): *δ* 173.2, 172.8, 172.4 (3C, C=O, Pro-2, Pro-1 and Ile), 171.2, 169.0, 168.5 (3C, C=O, Phe, Pro-3 and Leu), 167.8 (C=O, Val), 152.4 (C=O, Boc), 132.7 (*γ*-C, Phe), 130.9 (2C, *m*-C’s, Phe), 129.1 (2C, *o*-C’s, Phe), 128.2 (*p*-C, Phe), 79.8 (*α-*C, Boc), 56.5, 55.9 (2C, *α*-C’s, Pro-2 and Pro-3), 55.4 (*α*-C, Ile), 55.0 (*α-*C, Pro-1), 54.2 (OCH_3_), 53.4, 50.6 (2C, *α*-C’s, Phe and Val), 48.4, 47.5, 47.2 (3C, *δ*-C’s, Pro-1–3), 46.8 (*α*-C, Leu), 38.3, 37.6 (2C, *β*-C’s, Phe and Ile), 37.0, 29.6 (2C, *β*-C’s, Leu and Val), 28.4 (3C, *β*-C’s, Boc), 26.5, 26.2, 25.9 (3C,* β*-C’s, Pro-1–3), 25.6 (*γ*-C, Ile), 24.2, 23.9, 23.5 (3C, *γ*-C’s, Pro-1–3), 23.2 (*γ*-C, Leu), 22.1 (2C, *δ*-C’s, Leu), 18.3 (2C, *γ*-C’s, Val), 14.9 (*γ’*-C, Ile), 9.3 (*δ*-C, Ile) ppm; Anal. Calcd. for C_47_H_73_N_7_O_10_: C, 62.99; H, 8.21; N, 10.94. Found: C, 62.97; H, 8.19; N, 10.95%.

*Synthesis of cyclic heptapeptide, rolloamide A [* ***8*** *]*

To synthesize compound **8**, linear heptapeptide unit **7 **(0.005 mol) was deprotected at carboxyl end using LiOH (0.18 g, 0.0075 mol) to get Boc-Phe-Pro-Val-Pro-Leu-Pro-Ile-OH. The deprotected heptapeptide unit (0.005 mol) was now dissolved in CHCl_3 _(50 mL) at 0 °C. To the above solution, pentafluorophenol / *p*-nitrophenol (1.23 g/0.94 g, 0.0067 mol) and DCC (1.06 g, 0.005 mol) was added and stirred at RT for 12 h. The reaction mixture was filtered and the filtrate was washed with 10% NaHCO_3_ solution (2 × 25 mL) and 5% HCl (3 × 15 mL) to get the corresponding pentafluorophenyl/*p*-nitrophenyl ester Boc-Phe-Pro-Val-Pro-Leu-Pro-Ile-O-*pfp*/Boc-Phe-Pro-Val-Pro-Leu-Pro-Ile-O-*pnp*. To this compound (0.004 mol) dissolved in chloroform (25 mL), trifluoroacetic acid (0.91 g, 0.008 mol) was added, stirred at RT for 1 h, and washed with 10% NaHCO_3 _solution (3 × 20 mL). The organic layer was dried over anhydrous Na_2_SO_4 _to get Phe-Pro-Val-Pro-Leu-Pro-Ile-O-*pfp*/Phe-Pro-Val-Pro-Leu-Pro-Ile-O-*pnp* which was dissolved in CHCl_3 _(25 mL) and TEA/NMM/pyridine (2.8 mL/2.21 mL/1.61 mL, 0.02 mol) was added. Then, whole content was kept for 1 week time at 0 °C. The reaction mixture was washed with 10% NaHCO_3_ and 5% HCl solutions (3 × 25 mL). The organic layer was dried over anhydrous Na_2_SO_4_. Finally, chloroform was distilled off and crude cyclized product was crystallized from CHCl_3_/*n*-hexane to get pure cyclo (phenylalanyl-prolyl-valyl-prolyl-leucyl-prolyl-isoleucyl) [**8**] as white needles. 

*Biological activity*

*Antimicrobial activity studies*

The synthesized cyclic heptapeptide was subjected to antibacterial and antifungal activity studies using Modified Kirby-Bauer’s method (48) against strains *Bacillus subtilis (B. subtilis)*,* Staphylococcus aureus (S. aureus)*,* Pseudomonas aeruginosa (P. aeruginosa), Klebsiella pneumoniae (K. pneumoniae) *and *Megascoplex audouinii (M. audouinii)*, *Trichophyton mentagrophytes (T. mentagrophytes)*, *Candida albicans (C. albicans)* and *Aspergillus niger* *(A. niger)* at 25-6 μg mL^–1^ concentration. 

MIC values of test compound were determined by tube dilution technique. Synthesized peptide **8** was dissolved in sterile DMF to prepare a stock solution of 1 mg/mL. Stock solution was aseptically transferred and suitably diluted with sterile broth medium to contain seven different concentrations of test compound ranging from 200-6 *μ*g mL^–1^ in different test tubes. All the tubes were inoculated with one loopful of one of the test bacterium/fungi. The process was repeated with different test bacteria/fungi. Tubes inoculated with bacterial cultures were incubated at 37 °C for 18 h and the presence/absence of growth of the bacteria was observed. In case of antifungal activity, DMSO was used instead of DMF and the tubes inoculated with fungal cultures were incubated at 37 °C for 48 h.

From these results, MIC of synthesized peptide was determined against each test bacterium/fungi. A spore suspension in sterile distilled water was prepared from 5 days old culture of the test bacteria/fungi growing on nutrient broth media/sabouraud’s broth media. About 20 mL of the growth medium was transferred into sterilized petri plates and inoculated with 1.5 mL of the spore suspension (spore concentration – 6 × 10^4^ spores mL^–1^). Filter paper disks of 6 mm diameter and 2 mm thickness were sterilized by autoclaving at 121 °C for 15 min. Each petri plate was divided into five equal portions along the diameter to place one disc. Three discs of test sample were placed on three portions together with one disc with reference drugs ciprofloxacin and griseofulvin and a disk impregnated with the solvent (DMF/DMSO) as negative control. Test sample and the reference drugs were tested at the same concentration of 25-6 μg mL^–1^. The petri plates inoculated with bacterial/fungal cultures were incubated at 37 °C for 18 h and 48 h, respectively. Diameters of the zones of inhibition (ZOI in mm) were measured and the average diameters for test sample were calculated of triplicate sets. The diameters obtained for the test sample were compared with that produced by the standard drugs - ciprofloxacin and griseofulvin. 

*Anthelmintic activity studies*

Anthelmintic activity studies were carried out using Garg’s method against different species of earthworms like *Megascoplex konkanensis (M. konkanensis)*, *Pontoscotex corethruses (P. corethruses)*, and *Eudrilus* species at 2 mg mL^−1^ concentration (49). Suspension of sample was prepared by triturating synthesized peptide (200 mg) with tween 80 (0.5%) and distilled water and the resulting mixture was stirred using a mechanical stirrer for 30 min. The suspension was diluted to contain 0.2% *w/v *of the test sample. Suspensions of the reference drugs, mebendazole, and piperazine citrate were prepared with the same concentration in a similar way. Three sets of five earthworms of almost similar sizes (2 inch in length) were placed in petri plates of 4 inch diameter containing 50 mL of suspension of test sample and reference drug at RT. Another set of five earthworms was kept as control in 50 mL suspension of distilled water and tween 80 (0.5%). The paralyzing and death times were noted and their mean was calculated for triplicate sets. 

Detailed experimental procedures of antimicrobial and anthelmintic activity studies are given in our previously published reports (50-55).

## Results

*Chemistry*

**Figure 1:**
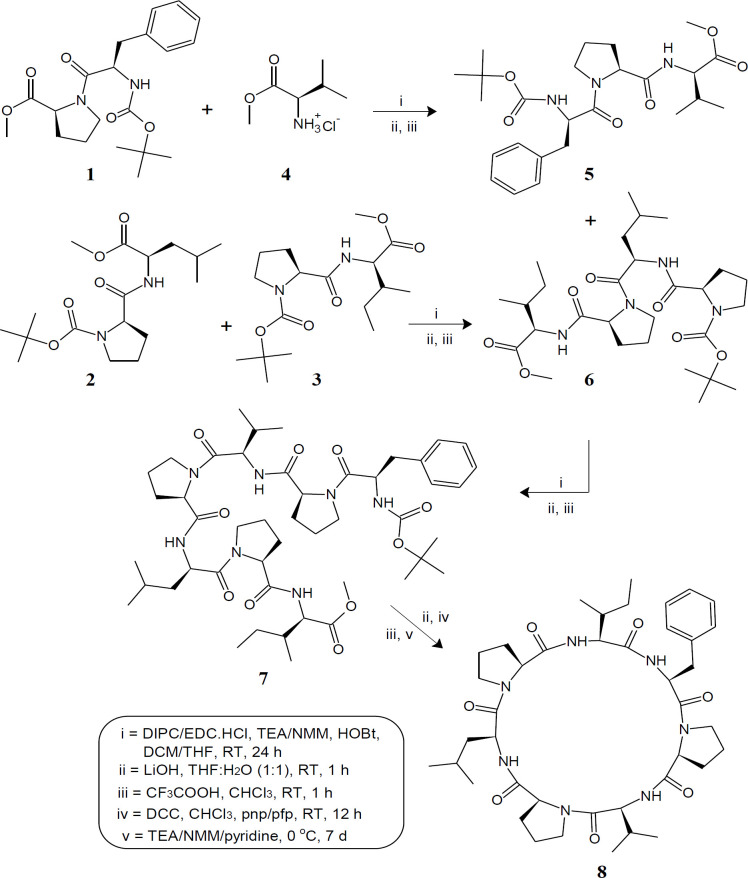
Synthetic route for rolloamide A [8].

In the present investigation, the first total synthesis of rolloamide A was accomplished using disconnection strategy (56). The cyclic heptapeptide molecule was split into three dipeptide units Boc-Phe-Pro-OMe [**1**], Boc-Pro-Leu-OMe [**2**], Boc-Pro-Ile-OMe [**3**] and also an amino acid methyl ester hydrochloride Val.OMe.HCl [**4**]. The required dipeptide units **1-3** were prepared by coupling of Boc-amino acids *viz. *Boc-Phe and Boc-Pro with corresponding amino acid methyl ester hydrochlorides such as Pro-OMe.HCl, Leu-OMe.HCl, and Ile-OMe.HCl employing EDC.HCl as a coupling agent. Ester group of dipeptide **1** was removed by alkaline hydrolysis with LiOH and coupled with Val.OMe.HCl to get tripeptide unit Boc-Phe-Pro-Val-OMe [**5**]. Ester group of dipeptide **2** was removed by alkaline hydrolysis with LiOH and Boc group of dipeptide **3** was removed by using trifluoroacetic acid. Both the deproptected dipeptides were coupled together using DIPC as coupling agent and TEA as base, to get the tetrapeptide unit Boc-Pro-Leu-Pro-Ile-OMe [**6**]. Similarly, tripeptide **5** after deprotection at carboxyl end, was coupled with tetrapeptide **6** deprotected at amino terminal, to get the linear heptapeptide unit Boc-Phe-Pro-Val-Pro-Leu-Pro-Ile-OMe [**7**]. The ester group of linear fragment was removed using LiOH and pentafluorophenyl (*pfp*) / *p*-nitrophenyl (*pnp*) ester group was introduced. The Boc-group was removed using CF_3_COOH and deprotected linear fragment was now cyclized by keeping the whole contents at 0 °C for 7 days in the presence of catalytic amount of TEA/NMM/pyridine to get cyclic compound **8 **(Figure 1). The structures of the newly synthesized cyclic heptapeptide as well as intermediates linear di/tri/tetra/heptapeptides were confirmed by IR, ^1^H NMR, ^13^C NMR, as well as elemental analysis. In addition, mass spectra were recorded for the synthesized cycloheptapeptide.

*Cyclo (phenylalanyl-prolyl-valyl-prolyl-leucyl-prolyl-isoleucyl)* *[****8****]*

 m.p. 196-198 °C (d), Yield 83.6% (TEA), 76.3% (NMM), 69.5% (C_5_H_5_N), [*α*]_D_: –86.3° (–86.5°) (*c *0.93, MeOH); R_f_ - 0.75; IR (KBr):* v *3128-3125, 3119 (m, -NH str, amide), 3066, 3062 (w, -CH str, aromatic ring), 2999-2995, 2992-2986 (m, -CH str, cyclic CH_2_ and CH), 2966, 2929-2925 (m, -CH str, asym, CH_3 _and CH_2_), 2867, 2861, 2846 (m, -CH str, sym, CH_3_ and CH_2_), 1674-1669, 1639-1634 (s, -C=O str, 3° and 2° amide), 1556, 1429 (m, skeletal bands, aromatic ring), 1527-1523 (m, -NH bend, 2° amide), 1379, 1357 (m, -CH bend, *iso*-propyl group), 717, 685 (s, -CH bend, oop, aromatic ring); ^1^H NMR (300 MHz, CDCl_3_): *δ* 10.25 (1H, br. s, -NH, Phe), 9.94 (1H, br. s, -NH, Leu), 9.69 (1H, br. s, -NH, Ile), 7.29 (2H, t, *J* = 7.3 Hz, *m*-H’s, Phe), 7.22 (2H, d, *J* = 7.25 Hz, *o*-H’s, Phe), 7.19 (1H, t, *J* = 7.4 Hz, *p*-H, Phe), 7.14 (1H, br. s, -NH, Val), 5.25 (1H, q, *J* = 5.75 Hz, *α*-H, Phe), 5.16 (1H, t, *J* = 7.8 Hz, *α*-H, Val), 4.95 (1H, t, *J* = 7.75 Hz, *α*-H, Pro-3), 4.90 (1H, t, *J* = 7.8 Hz, *α*-H, Pro-2), 4.75 (1H, q, *J* = 6.75 Hz, *α*-H, Leu), 4.59 (1H, t, *J* = 7.7 Hz, *α*-H, Ile), 3.98 (2H, t, *J* = 7.8 Hz, *δ*-H’s, Pro-2), 3.94 (1H, t, *J* = 8.25 Hz, *α*-H, Pro-1), 3.79 (2H, t, *J* = 7.9 Hz, *δ*-H’s, Pro-1), 3.75 (2H, t, *J* = 7.9 Hz, *δ*-H’s, Pro-3), 3.11 (2H, d, *J* = 5.85 Hz, *β*-H’s, Phe), 2.79-2.74 (2H, m, *β*-H’s, Pro-3), 2.51-2.44 (1H, m, *β*-H, Ile), 2.26-2.22 (1H, m, *γ*-H, Leu), 2.19-2.13 (1H, m, *β*-H’s, Val), 2.05-1.94 (4H, m, *β*-H’s, Pro-1–2), 1.88-1.83 (2H, m, *γ*-H’s, Pro-1), 1.79 (2H, t, *J* = 6.85 Hz, *β*-H’s, Leu), 1.73-1.64 (4H, m, *γ*-H’s, Pro-2–3), 1.33-1.28 (2H, m, *γ*-H’s, Ile), 1.20 (3H, d, *J* = 6.55 Hz, *γ’*-H’s, Ile), 1.12 (6H, d, *J* = 6.6 Hz, *δ*-H’s, Leu), 1.07 (6H, d, *J* = 6.65 Hz, *γ*-H’s, Val), 0.95 (3H, t, *J* = 7.25 Hz, *δ*-H’s, Ile) ppm; ^13^C NMR (CDCl_3_, 300 MHz): *δ* 173.7, 173.4, 173.1 (3C, C=O, Pro-2, Phe and Pro-3), 172.3, 172.0, 171.8 (3C, C=O, Pro-1, Ile and Leu), 170.4 (C=O, Val), 136.9 (*γ*-C, Phe), 130.4 (2C, *o*-C’s, Phe), 129.5 (2C, *m*-C’s, Phe), 127.7 (*p*-C, Phe), 62.7, 62.0 (2C, *α*-C’s, Pro-1 and Pro-3), 60.3 (*α*-C, Ile), 59.0 (*α*-C, Pro-2), 55.9 (*α-*C, Val), 53.1, 52.9 (2C, α-C’s, Phe and Leu), 48.0, 47.6, 47.2 (3C, *δ*-C’s, Pro-1–3), 39.7, 39.4 (2C, *β*-C’s, Phe and Leu), 35.2, 34.9 (2C,* β*-C’s, Ile and Val), 32.9, 32.6, 30.8 (3C, *β*-C’s, Pro-1–3), 26.8 (*γ*-C, Ile), 25.7 (*γ*-C, Leu), 24.5 (2C, *δ*-C’s, Leu), 22.9, 22.6, 23.0 (3C, *γ*-C’s, Pro-1–3), 18.5 (2C, *γ*-C’s, Val), 16.6 (*γ’*-C, Ile), 11.3 (*δ*-C, Ile) ppm; FAB MS: *m/z* 764.6 (M + H)^+^, 736.6 (764.5–CO)^+^, 667.6 (Val-Pro-Leu-Pro-Ile-Phe / Leu-Pro-Ile-Phe-Pro-Val)^+^, 651.6 (Phe-Pro-Val-Pro-Leu-Pro)^+^, 639.5 (667.6–CO)^+^, 623.5 (651.6–CO)^+^, 617.6 (Pro-Val-Pro-Leu-Pro-Ile)^+^, 589.5 (617.6–CO)^+^, 568.5 (Leu-Pro-Ile-Phe-Pro)^+^, 554.5 (Phe-Pro-Val-Pro-Leu)^+^, 540.5 (568.5–CO)^+^, 526.5 (554.5–CO)^+^, 520.5 (Val-Pro-Leu-Pro-Ile)^+^, 504.5 (Pro-Val-Pro-Leu-Pro)^+^, 492.5 (520.5–CO)^+^, 476.5 (504.5–CO)^+^, 471.5 (Leu-Pro-Ile-Phe)^+^, 443.5 (471.5–CO)^+^, 441.5 (Phe-Pro-Val-Pro)^+^, 413.5 (441.5–CO)^+^, 407.5 (Val-Pro-Leu-Pro / Pro-Val-Pro-Leu)^+^, 379.5 (407.5–CO)^+^, 344.4 (Phe-Pro-Val)^+^, 324.4 (Leu-Pro-Ile)^+^, 316.4 (344.4–CO)^+^, 310.4 (Val-Pro-Leu)^+^, 296.4 (324.4–CO)^+^, 294.4 (Pro-Val-Pro)^+^, 282.4 (310.4–CO)^+^, 266.4 (294.4–CO)^+^, 245.2 (Phe-Pro)^+^, 217.2 (245.2–CO)^+^, 211.2 (Leu-Pro)^+^, 197.2 (Val-Pro / Pro-Val)^+^, 183.2 (211.2–CO)^+^, 169.2 (197.2–CO)^+^, 148.1 (Phe)^+^, 120.1 (Phe immonium ion), 114.1 (Leu)^+^, 100.1 (Val)^+^, 98.1 (Pro)^+^, 91.1 (C_7_H_7_)^+^, 86.1 (Leu/Ile immonium ion); 77.1 (C_6_H_5_)^+^, 72.1 (Val immonium ion); 70.1 (Pro immonium ion), 57.1 (C_4_H_9_)^+^, 43.1 (C_3_H_7_)^+^, 29.1 (C_2_H_5_)^+^, 15.0 (CH_3_)^+^ ppm; Anal. Calcd. for C_41_H_61_N_7_O_7_: C, 64.46; H, 8.05; N, 12.83. Found: C, 64.45; H, 8.08; N, 12.85%. 

*Pharmacology*

The synthesized cyclopeptide 8 was screened for *in-vitro* antimicrobial activity against gram-positive bacteria *B. subtilis* and *S. aureus*, gram-negative bacteria *P. aeruginosa* and *K. pneumoniae*, cutaneous fungi *M. audouinii *and *T. mentagrophytes*, diamorphic fungi *C. albicans* and *A. niger* and for anthelmintic activity against earthworms *M. konkanensis*,* P. corethruses *and *Eudrilus* sp. The results of pharmacological activity studies are tabulated in Table 1 and Table 2. Antibacterial, antifungal, and anthelmintic data were subjected to one-factor ANOVA using GraphPad InStat software. The *P*-values for antibacterial and anthelmintic data were 0.00554 and 0.00553, respectively which were considered very significant, and the variations among column means were significantly greater than expected by chance. However, in case of antifungal activity studies, the *P*-value was 0.414 which was not considered very significant, indicating that significant difference does not exist between the values of zone of inhibition (ZOI) of species tested (Table 3).

**Table 1:** Antimicrobial activity data for compound **8**

**Compd.**	**Diameter of zone of inhibition (mm)**							
	**Bacterial strains**				**Fungal strains**			
	***B.*** ***subtilis***	***S.*** ***Aureus***	***P.*** ***aeruginosa***	***K.*** ***pneumoniae***	***C.*** ***albicans***	***M.*** ***audouinii***	***A.*** ***niger***	***T.*** ***mentagrophytes***
**8**	9 (25)^a^	13 (25)	27 (6)	23 (12.5)	24 (6)	13 (6)	–	16 (6)
Control	–	–	–	–	–	–	–	–
Ciprofloxacin	20 (6)	19 (12.5)	25 (6)	20 (12.5)	–	–	–	–
Griseofulvin	–	–	–	–	20 (6)	19 (6)	18 (12.5)	21 (6)

^a^Values in brackets are MIC values ( g mL^–1^).

**Table 2:** Anthelmintic activity data for compound **8**.

**Compd.**	**Earthworm species**					
	***M. konkanensis***		***P. corethruses***		***Eudrilus sp.***	
	**Mean** ** paralyzing time (min)** ^a^	**Mean** **death time** **(min)**	**Mean ** **paralyzing time (min)**	**Mean** **death time** **(min)**	**Mean paralyzing time (min)**	**Mean** **death time** **(min)**
**8** ^b^	08.22 ± 0.31	10.06 ± 0.23	16.02 ± 0.44	17.59 ± 0.59	08.45 ± 0.43	10.25 ± 0.37
Control^c^	–	–	–	–	–	–
Mebendazole^b^	10.52 ± 0.62	12.57 ± 0.49	18.02 ± 0.58	19.49 ± 0.37	11.29 ± 0.40	13.37 ± 0.42
Piperazine citrate^b^	12.38 ± 0.49	13.55 ± 0.27	19.17 ± 0.44	22.17 ± 0.26	12.45 ± 0.19	13.44 ± 0.36

^a^Data are given as mean ± S.D. (n = 3); ^b^*c* = 2 mg mL^−1^; ^c^0.5% Tween 80 in distilled water.

**Table 3:** Summary of one-factor ANOVA for bioactivities

**Type of study**	**Source of variation**	**SS** ^*^	**df** ^#^	**MS** ^†^	**F**	***P*** **-value**	**F**_crit_
Antibacterial activity	Between groups	167	2	51.72	13.954	0.00554	5.143
	Within groups	85	6	3.71			
Antifungal activity	Between groups	181.375	2	60.46	1.21	0.414	6.591
	Within groups	200.5	6	50.13			
Anthelmintic activity	Between groups	103.44	2	51.72	13.95	0.00553	5.14
	Within groups	22.24	6	3.71			

^*^SS: sum of square; ^#^df: degree of freedom; ^†^MS: mean square.

## Discussion

Disappearance of absorption bands at 1748, 1269 cm^-1^, and 1387, 1368 cm^-1^ (C=O_str_ and C-O_str_, methyl ester group and C-H_bend_, *tert*-butyl group) in FT-IR spectrum of 8 clearly indicated cyclization of linear heptapeptide unit. This fact was further supported by disappearance of two singlets at *δ* 1.55 and 3.54, corresponding to protons of *tert*-butyl and methyl ester groups, in ^1^H NMR spectrum and disappearance of singlets at *δ* 79.8, 28.4, and 54.2, corresponding to carbon atoms of *tert*-butyl and methyl ester groups, in ^13^C NMR spectrum of 8. Six signals between *δ* 5.25-4.59 in the proton spectrum of 8 suggested a peptidic structure for the synthesized product, with these signals being attributable to the *α*-protons of all amino acid units. The ^1^H NMR spectrum of cyclized product showed the presence of four broad singlets between *δ* 10.25-9.69 and 7.14 corresponding to the imino protons of the phenylalanine, leucine, isoleucine, and valine moieties, remaining amino acids being three proline units, indicating similarity of the structure of the newly synthesized cycloheptapeptide with the natural molecule. Moreover, ^1^H/^13^C NMR spectra of the cyclized product 8 showed characteristic peaks confirming the presence of all the 61 protons and 41 carbon atoms. Presence of pseudomolecular ion peak at *m/z* 764.6 corresponding to the molecular formula C_41_H_61_N_7_O_7_ in mass spectrum of 8, along with other fragment ion peaks resulting from cleavage at ‘Pro-Val’, ‘Pro-Leu’, ‘Ile-Phe’, and ‘Phe-Pro’ amide bond levels, showed exact sequence of attachment of all the seven amino acid units in a chain. In addition, elemental analysis data of 8 afforded values (±0.03) strictly in accordance to the molecular composition. 

Polypeptide 8 displayed significant antimicrobial activity against pathogenic fungi *C. albicans* (ZOI: 24 mm, MIC: 6 *μ*g/mL) and Gram-negative bacteria *P. aeruginosa* (ZOI: 27 mm, MIC: 6 *μ*g/mL) and *K. pneumoniae* (ZOI: 23 mm, MIC: 12.5 *μ*g/mL), which was even better than standard drugs – griseofulvin (ZOI: 20 mm, MIC: 6 *μ*g/mL) and ciprofloxacin (ZOI: 25 mm, MIC: 6 *μ*g/mL/ZOI: 20 mm, MIC: 12.5 *μ*g/mL). Moreover, compound 8 (ZOI: 13-16 mm, MIC: 6 *μ*g/mL) showed moderate activity against dermatophytes, in comparison to reference compound – griseofulvin (ZOI: 19-21 mm, MIC: 6 *μ*g/mL). The activity showed by polypeptide 8 against Gram-positive bacteria *B. subtilis* and *S. aureus* was not found to be significant (ZOI: 9-13 mm, MIC: 25 *μ*g/mL) and no activity was observed against fungus *Aspergillus niger*. 

Analysis of anthelmintic activity data suggested that newly synthesized polypeptide 8 (MPT/MDT ratio – 8.22-16.02/10.06-17.59 min) showed better activity against all three earthworm species, in comparison to standard drug - mebendazole (MPT/MDT ratio – 10.52-18.02/12.57-19.49 min) and piperazine citrate (MPT/MDT ratio – 12.38-19.17/13.44-22.17 min). *Megascoplex konkanensis* species was found to be less sensitive towards the newly synthesized polypeptide 8, in comparison to other two earthworm species. 

## Conclusion

The synthesis of natural cyclopolypeptide 8 was carried out successfully and the yield varied according to the base (TEA/NMM/pyridine) used for cyclization ranging from 69.5% to 83.6%. Further, pentafluorophenyl ester method utilizing TEA was proved to be an effective with maximum yield of 83.6%, for cyclization of linear heptapeptide fragment. Newly synthesized cyclopeptide was found to exhibit good level of activity against pathogenic *Candida* sp. and Gram-negative bacteria. Overall, gram-negative bacteria were found to be more sensitive towards the synthesized peptide, in comparison to gram-positive bacteria. Further, significant activity was observed against against earthworm species, in comparison to standard drugs. On passing toxicity tests, synthesized cyclopeptide 8 may prove good candidate for clinical studies and can be new antimicrobial and anthelmintic drug of future.
